# Th1 and Th2 epitopes of *Cowdria* polymorphic gene 1 of *Ehrlichia ruminantium*

**DOI:** 10.4102/ojvr.v90i1.2070

**Published:** 2023-03-23

**Authors:** Tlou A. Ngoepe, Alri Pretorius, Helena C. Steyn, Mirinda van Kleef

**Affiliations:** 1 Department of Immunology, Agricultural Research Council-Onderstepoort Veterinary Research, Pretoria, South Africa; 2 Department of Veterinary Tropical Diseases, Faculty of Veterinary Science, University of Pretoria, Pretoria, South Africa; 3 Department of Immunology, Faculty of Health Sciences, University of Pretoria, Pretoria, South Africa

**Keywords:** Heartwater, vaccine epitopes, PBMC, lymphocyte proliferation assay, ELISA, ELISpot, cytokine qRT-PCR, flow cytometry

## Abstract

*Cowdria* polymorphic gene 1 (cpg1, Erum2510, ERUM_RS01380) has been shown to induce 30% and 100% protection in sheep immunised by deoxyribonucleic acid (DNA) prime combined with DNA boost and DNA prime combined with protein boost, respectively, against heartwater infection via needle challenge. To localise its antigenic regions for inclusion in a multi-epitope DNA vaccine against heartwater, Erum2510 was cleaved into five overlapping subfragments. These subfragments were expressed individually in an *Escherichia coli* host expression system and evaluated for their ability to induce proliferative responses, Th1 and Th2 cytokines (interferon gamma [IFN-γ] and interleukin 4 [IL-4]) via enzyme-linked immunospot (ELISpot), quantitative real time polymerase chain reaction (qRT-PCR) and flow cytometry. Recombinant (*r*)proteins 3 and 4 were shown to induce immunodominant Th1 and Th2 immune responses characterised by the secretion of effector cytokines IFN-γ and IL-4 in addition to differential messenger ribonucleic acid (mRNA) expression of tumour necrosis factor (TNF), IL-2, IL-1, IL-18, IL-10, transforming growth factor (TGF), granulocyte-macrophage colony-stimulating factor (GM-CSF) and inducible nitric oxide synthase (iNOS). Thirty-seven overlapping synthetic peptides (16 mer) spanning the lengths of these immunodominant *r*proteins were synthesised and assayed. A peptide pool comprising p9 and p10 derived from *r*protein 3 induced a Th1-biased immune response. A peptide pool comprising p28 and p29 derived from rprotein 4 induced a mixed Th1 and Th2 immune response characterised by secretion of IFN-γ and differential mRNA expression of IL-1, IL-2, IL-10, IL-12, iNOS, TGF, TNF and GM-CSF. Only one of the peptides (p29) induced secretion of IL-4. Phenotypic analysis showed significant activation of cluster of differentiation 8+ (CD8+), cluster of differentiation 4+ (CD4+) and B+ lymphocyte populations. Findings suggest that Erum2510 *r*proteins and synthetic peptides can induce both cellular and humoral immune responses, thereby implicating their importance in protection against heartwater.

**Contribution:**

This study will facilitate the design of an effective multi-epitope DNA vaccine against heartwater that will contribute to control this economically important disease in sub-Saharan Africa and beyond.

## Introduction

Heartwater is an infectious, noncontagious and lethal tick-borne disease of domestic and some wild ruminants (Dumler et al. 2001) that is characterised by high fever, nervous signs, oedema of the heart and lungs and eventually death. The causative agent of heartwater is a *Rickettsia*, *Ehrlichia ruminantium*, which is transmitted by ticks of the genus *Amblyomma* (Camus et al. 1996). It is regarded as an economically important disease that causes stock losses in sub-Saharan Africa, thereby threatening food security (Uilenberg 1982).

Currently, control of the disease relies mainly on the use of acaricides, antibiotic treatment (Peregrine 1994) and the commercial, infection and treatment blood vaccine (Van der Merwe 1987). The latter has several drawbacks, as it requires that the infective blood be kept on dry ice and thawed immediately before inoculation; it requires treatment with antibiotics; and it does not protect against all isolates (Allsopp 2009). Therefore, there is a need to develop a vaccine that is safe, affordable, effective and does not require transport on dry ice, as most vaccines do still require a cold chain.

To date, a number of experimental heartwater vaccines – for example, inactivated, attenuated and deoxyribonucleic acid (DNA) – have been developed, and all have demonstrated varying levels of inadequate protection against a natural field challenge (Collins et al. 2003). When the humoral major antigenic protein (MAP1) of *E. ruminantium* was used as a DNA prime, recombinant protein boost vaccine in mice, it induced variable protection (13% – 67%) against virulent needle challenge, characterised by a cellular and humoral immune response (Nyika et al. 2002). However, there are no reports that this vaccine was tested in sheep, goats or cattle.

The 1H12 cocktail DNA vaccine containing four *E. ruminantium* genes provided 100% protection against a virulent needle challenge in sheep (Collins et al. 2003) but only 20% protection in sheep to a natural tick challenge in the field (Pretorius et al. 2008). Thereafter, selected 1H12 epitopes and multiple-vaccine candidate *E. ruminantium* epitopes that induce varied cellular immune responses (Thema et al. 2019a, 2019b) were combined in a multi-epitope DNA vaccine. This vaccine induced 60% protective efficacy in sheep after a laboratory tick challenge (Tshilwane et al. 2019).

It was traditionally thought that protective immunity against *E. ruminantium* is not antibody related because antiserum from heartwater immune cattle was not able to passively transfer protective immunity (Du Plessis 1970). However, various serological tests and Western blot analysis of serum from heartwater immune animals revealed the presence of *E. ruminantium-*specific antibodies (Rossouw et al. 1990). Mwangi and coworkers (1998) later showed that *E. ruminantium* immune cattle elicited a predominant cellular immune response characterised in vitro by CD4+, CD8+ and γδ+ T cells, and the cytokines interleukin 2 (IL-2), interferon gamma (IFN-γ), tumour necrosis factor (TNF)-α and TNF-β. In contrast, a minor B cell response suggestive of an antibody response was also detected. The function of antibodies in protective immunity against heartwater is still unknown, and it may be involved in opsonisation, complement-mediated killing and/or antibody-dependent cell-mediated cytotoxicity (Totté et al. 1999). Because a protective immune response to *E. ruminantium* infection has both cellular and humoral components, perhaps a subunit vaccine that includes both will result in improved protection. Thus, the effectiveness of the multi-epitope DNA vaccine may be further improved by combining well-defined specific pathogen epitopes that are able to trigger both humoral and cellular immunity (Hajissa et al. 2019).

Erum2510 (ERUM_RS01380) is a previously identified polymorphic gene of *E. ruminantium* (Perez et al. 1997) and referred to as *Cowdria* polymorphic gene 1 (CpG1). It was shown to induce 100% protective immunity in ruminants as a heterologous DNA prime and protein boost vaccine against a needle challenge (Pretorius et al. 2010). Antigen-specific lymphocyte proliferation was detected, as well as antibodies directed to *r*Erum2510 after immunisation. Based on these results, it was hypothesised that Erum2510 contains Th1 and Th2 epitopes that could confer optimal protective immunity when included in the multi-epitope DNA vaccine against heartwater. Therefore, this study reports the use of epitope mapping to identify Erum2510 epitopes that induce protective immune responses characterised by CD8+, CD4+ and B+ cells and the expression of Th1 and Th2 cytokines.

## Methods and materials

### Cloning of Erum2510 gene subfragment

*E. ruminantium* (Welgevonden) was grown in bovine endothelial cells (Zweygarth & Josemans 2001) and genomic DNA prepared by Percoll density gradient and DNeasy blood and tissue kit (QIAGEN) as described previously (Mahan et al. 1995). For ease of cloning an adenine (A) and thymine (T) rich gene sequence, Erum2510 was cleaved into six overlapping subfragments, for which amplicon specific primers were designed (Appendix 1). To facilitate directional cloning, a CACC nucleotide sequence was added to the 5′ end of the forward primer.

The DNA subfragments were amplified in a 25 μL reaction containing 31 ng *E. ruminantium* (Welgevonden) genomic DNA, 1 × Q5 high-fidelity master mix (New England BioLabs^®^ Inc.) and 0.4 μM forward and 0.4 μM reverse primers (Inqaba Biotec). The DNA subfragments were each polymerase chain reaction (PCR) amplified using the following cycling parameters: initial denaturation for 2 min at 95 °C, followed by 30 cycles of denaturation at 95 °C for 30 s, annealing at 55 °C for 30 s, extension at 72 °C for 3 min and a final extension at 72 °C for 7 min, using a GeneAmp^®^ PCR 9700 amplifier (Applied Biosystems). Amplicons were cloned in the pET102 directional TOPO^®^ vector (Invitrogen) following the protocol of the manufacturer. Clones containing inserts of the correct size were sequenced at Inqaba Biotec (Pretoria, South Africa) using the T7 reverse primer and TrxFus forward primer (1 μMol) to ensure that the sequence was correct and that the his-tag was in frame.

### Expression and purification of *r*Erum2510

Recombinant proteins were expressed in the BL21 (DE3) strain of *Escherichia coli* (*E. coli*) as thioredoxin, his-tagged fusion proteins, using the Overnight Express™ Instant Terrific Broth media (Gershoni et al. 2007) (Novagen^®^), as described previously (Thema et al. 2016). Proteins were extracted using the BugBuster™ Protein Extraction Reagent, following the protocol of the manufacturer (Novagen^®^) and recombinant protein purified using Protino^®^ Ni-TED 150 packed columns, following instructions of the manufacturer.

Recombinant proteins were analysed using sodium dodecyl sulphate – polyacrylamide gel electrophoresis (SDS-PAGE) and anti-His (Roche) Western blot analysis according to standard protocols. Briefly, the purified *r*proteins were separated on a 12% Criterion PGX™ precast SDS-PAGE gel (Bio-Rad) and transferred to a polyvinylidene fluoride (PVDF) membrane. The membrane was incubated with anti-His6 antibodies (1:6000 dilution) (Roche), followed by a secondary antibody (goat anti-mouse immunoglobulin G [IgG], 1 in 20 000 dilution), and the protein bands were visualised using BM Blue peroxidase (POD) precipitating substrate (Roche).

#### Peptide synthesis

A total of thirty-seven 16-mer peptides (overlapping by 8 amino acids, Figure 1-A1) were designed, spanning the length of Erum2510 subfragments 3 (peptides 1–19) and 4 (peptides 20–37). They were synthesised by GenScript USA Inc., New Jersey, United States (US).

### Experimental animals

#### Immune animals

Two merino sheep (sM2 and s6823) were bought from a heartwater-free area in the Free State province, South Africa, where the *Amblyomma hebraeum* tick is absent. They tested negative for *E. ruminantium*, using the pCS20 quantitative real-time polymerase chain reaction (qRT-PCR), before the experiment began (Steyn et al. 2008). Sheep were infected with heartwater (Welgevonden strain)–infected ticks. For the primary infection, 10 adult heartwater-infected ticks were allowed to feed on the naïve sheep. Sheep were monitored daily for disease symptoms (body temperature above 41.5 °C, loss of appetite, heavy breathing, depression, hanging head, stiff gait, exaggerated blinking, chewing movements, anorexia and signs of nervous symptoms). Sheep were treated using Terramycin^®^100 (1 mL/10 kg body weight) after onset of the febrile response. Both animals then received an infected tick challenge, similar to the primary infection, 30 days after antibiotic treatment and were monitored daily for onset of clinical symptoms (Thema et al. 2016). Heartwater infection of sheep (approximately 12 days after primary infection) and ticks (before sheep infection) was confirmed by pCS20 qRT-PCR (Steyn et al. 2008). The severity of infection was determined by scoring the clinical signs according to a reaction index (RI). These animals were scored according to their temperature reaction and symptoms displayed, as well as treatment received (Pretorius et al. 2007).

#### Peripheral blood mononuclear cell isolation

Blood (50 mL per sheep) was collected in BD Vacutainer^®^ – ethylenediaminetetraacetic acid (EDTA) (Beckton, Dickson) tubes. Peripheral blood mononuclear cells (PBMCs) were isolated by density gradient centrifugation using Ficoll-Histopaque^®^-1077 (Sigma Aldrich^®^), as instructed by the manufacturer. Peripheral blood mononuclear cells were re-suspended in 1 mL complete Roswell Park Memorial Institute 1640 media (cRPMI) containing 25 mM 4-(2-hydroxyethyl)-1-piperazineethanesulfonic acid (HEPES), 10% foetal calf serum (FCS), 2 mM L-glutamate, 5 × 10^-5^ M mercaptoethanol, PenStrep (0.2 mg/mL, streptomycin sulphate, 0.1 mg/mL, sodium benzylpenicillin [Invitrogen]) and cells counted using the TC1O Automated cell counter (Bio-Rad).

### Immunological assays

#### Lymphocyte proliferation assay

The lymphocyte proliferation assay (LPA) was carried out in triplicate in a 96-well half-area plate in a total volume of 100 μL/well (Costar), as described previously (Sebatjane et al. 2010). Peripheral blood mononuclear cells (4 × 10^6^ cells/mL) were stimulated with recombinant proteins and/or synthetic peptides (10 μg/mL). Crude Welgevonden antigen prepared from cell culture (10 μg/mL), as described previously (Van Kleef et al. 2002), and Concanavalin A (ConA, 10 μg/mL) were used as positive controls and unstimulated PBMCs as negative control. Cells were incubated at 37 °C, with 5% CO_2_ for five days, and proliferation was determined by measuring the incorporation of 0.5 μCi of [methyl-3H]thymidine (Sigma-Aldrich^®^) added during the final 18 h of the assay using a Liquid Scintillation & Luminescence counter (Trilux). Results were expressed as the stimulation index (SI) calculated by the counts per minute (cpm) of the test antigen (averaged from triplicate wells) divided by the mean cpm of the unstimulated PBMC. A response was considered positive if two times higher than the SI of unstimulated PBMC with a significant *p*-value of ≤ 0.05.

#### Enzyme-linked immunospot assay (interferon gamma and interleukin 4)

The expression of IFN-γ and interleukin-4 (IL-4) was monitored using the bovine, ovine and equine IFN-γ or IL-4 enzyme-linked immunospot (ELISpot™) kit (Mabtech) according to instructions of the manufacturer and as described previously (Liebenberg et al. 2012). Briefly, the IFN-γ or IL-4 monoclonal antibody–coated ELISpot plates (Millipore) were incubated with PBMC (2 × 10^5^ cells per well) in triplicate with Erum2510 *r*proteins and/or synthetic peptides (10 μg/mL). Welgevonden antigen (10 μg/mL) and ConA (10 μg/mL) as positive controls and unstimulated PBMC as negative control. The plates were developed after 48 h incubation at 37 °C in a humidified 5% CO_2_ incubator. Results are represented as spot-forming cells (SFC) per million, and the number of SFC produced after stimulation with antigen was compared with the number of SFC with no stimulation. Results were considered positive if the SFC of stimulated cells was 2× higher than SFC of unstimulated cells with a *p* ≤ 0.05. Spot-forming cells were enumerated using an automated KS ELISPOTAX10 reader (Zeiss).

#### Cytokine profiling using quantitative real time polymerase chain reaction

Cytokine profiling was carried out using qRT-PCR as described previously (Liebenberg et al. 2012). Peripheral blood mononuclear cells (4 × 10^6^/mL) were stimulated with 10 μg/mL *r*protein and/or synthetic peptides overnight (18 h) at 37 °C in a humidified incubator at 5% CO_2_. Stimulated PBMC were harvested and resuspended in 1 mL Trizol reagent (Sigma Aldrich^®^). The total ribonucleic acid (RNA) was isolated according to the TRI^®^-Reagent protocol, and genomic DNA contamination was removed using the QuantiTect Reverse Transcription Kit (QIAGEN) according to the instructions of the manufacturer. The rotor-gene SYBR green qRT-PCR kit (QIAGEN) was used to quantify cytokine RNA targets using the Rotor-gene Q (Qiagen). Ovine qRT-PCR primers for housekeeping genes glyceraldehyde 3-phosphate dehydrogenase (GAPDH) and β-actin, and cytokines IL-4, IL-1α, transforming growth factor (TGF)-β, granulocyte-macrophage colony-stimulating factor (GM-CSF), IL-10, IL-12, IL-18, TNF-α, IL-2 and inducible nitric oxide synthase (iNOS) (Table S1) were synthesised by Inqaba Biotec. The cycling conditions were set as follows: 5 min at 95 °C, 40 cycles of two-step cycling at 95 °C for 5 s and 60 °C for 10 s. Glyceraldehyde 3-phosphate dehydrogenase and β-actin are common housekeeping genes that were used to normalise cytokine gene expression. Gene expression was measured by relative quantitation using the 2^-ΔΔCt^ method (Livak & Schmittgen 2001). One fold increase (FI) indicates that the cytokine messenger ribonucleic acid (mRNA) concentration of the stimulated sample is twice that of nonstimulated samples, and the cytokines were considered to be upregulated if the mRNA increase difference was FI > 1 compared with both housekeeping genes.

#### Phenotypic analysis by flow cytometry

Peripheral blood mononuclear cells were incubated in triplicate with all of the individual Erum2510 *r*proteins (10 µg/mL) or peptides (10 µg/mL), ConA (10 μg/mL, positive control) and unstimulated PBMC (negative control), for 72 h at 37 °C in a humidified incubator at 5% CO_2_. The resulting cells were surface stained and intracellular cytokine stained, as described previously (Thema et al. 2016).

**Surface staining:** Cells were incubated for 15 min at room temperature with primary monoclonal antibodies recognising CD4 (immunoglobulin M [IgM], cell line GC50A), CD8 (IgG1, cell line CACT80C), CD45RO (immunoglobulin G3 [IgG3], cell line GC44A) or B cell (IgM, cell line BAQ44A) markers (Washington State University, Pullman, United States) at a dilution of 1:100 in phosphate-buffered saline (PBS) containing 0.5% FCS, 0.09% sodium azide. After two washes, secondary goat antimouse antibodies specific to IgM-APC (Serotec), IgG1-PE (Serotec) and IgG3-FITC (Serotec) were diluted to 1:8, 1:30 and 1:10, respectively, and added to the cells. After 15 min incubation and two washes, the cells were fixed with 2% formaldehyde (except those for intracellular cytokine staining [ICS]). Flow cytometry data were collected with a Beckman Coulter FC500 flow cytometer and analysed using Kaluza Software version 1.2 (Beckman Coulter, Brea, California, United States).

**Intracellular cytokine staining:** Cells to be stained intracellularly for cytokines were treated with BD GolgiPlug™ protein transport inhibitor containing Brefeldin A (BD Biosciences™) 4 h before harvesting. After surface staining (SS), these cells were fixed and permeabilised using the BD Cytifix and Cytoperm^TM^ kit (BD Biosciences) according to instructions of the manufacturer. Thereafter, the cells were stained with mouse antibovine IFN-γ-Alexafluor488 (1:20, Celtic Diagnostics) by incubation for 30 min. The cells were then washed twice and flow cytometry data collected with a Beckman Coulter FC500 flow cytometer and analysed using Kaluza Software version 1.2.

### Statistical analysis

The difference of significance between LPA, ELISpot assay and phenotypic analysis results were determined by Student *t*-test; the difference was considered significant at a *p*-value of ≤ 0.05.

### Ethical considerations

All animal research was performed in accordance with the stipulations of the animal ethics committee at the ARC Onderstepoort Veterinary Research, the University of Pretoria Animal Use and Care Committee (V096-13), and approved by the South African Department of Agriculture, Forestry and Fisheries (DAFF) Section 20 of the *Animal Diseases Act of 1984 (Act No 35 of 1984)*.

## Results

### Cloning of Erum2510 gene subfragments

Erum2510 was divided into six overlapping subfragments of 450 bp each, which were amplified by PCR using gene-specific primers. Four of these subfragments (fragments 3–6) were successfully cloned in the pET 102 directional TOPO^®^ expression vector, and this was confirmed by PCR using vector-specific primers (T7 reverse and TrxFus forward primer). Numerous attempts to clone PCR products of Erum2510 subfragment 1 and 2 individually failed, and as a result, these were cloned as one fragment of 900 bp encoding a predicted protein of 44.5 kDa.

### Expression and purification of Erum2510 *r*proteins in *E. coli*

The recombinant Erum2510 expressed subfragments 3–6 had the predicted size of 30 kDa each, with the exception of the two gene subfragments 1–2, which were cloned and expressed as one gene subfragment encoding a predicted protein of 45.5 kDa (Figure 2-A1). Three of the *r*proteins (subfragments 1–2, 4 and 5) were purified from the soluble supernatant, whereas *r*proteins translated from the gene subfragments 3 and 6 were purified from inclusion bodies. The protein concentration of the proteins ranged from 356 µg/mL to 1355 µg/mL.

### Lymphocyte proliferation assay with recombinant proteins

Two sheep (sM2 and s6823) were infected with *E. ruminantium* by tick feeding and treated when they developed heartwater symptoms of a febrile response. After the tick challenge, similar to the primary infection, both sheep were considered to be immune to *E. ruminantium* because they did not develop any heartwater disease symptoms. The ability of immune PBMC, collected from these sheep, to undergo clonal proliferation was evaluated with the LPA. Peripheral blood mononuclear cells from both sheep had significant and positive SI of 3 after stimulation with the crude Welgevonden-strain antigen. *r*Protein 3 showed a significant positive SI of 3 (sM2) and 10 (s6823), whereas *r*protein 4 had a significant positive SI of 4 (sM2) and 20 (s6823) (Figure 3-A1). *r*Protein 6 induced significant proliferation only in PBMC from s6823 (SI 4) (Figure S3).

### Enzyme-linked immunospot assays (interferon gamma and interleukin 4) with recombinant proteins

*r*Proteins 3 and 4 induced the highest secretion of IFN-γ and IL-4 in PBMC from s6823 (Figure 1a, c). However, the same Erum2510 *r*proteins failed to induce secretion of IL-4 in PBMC from sM2, although IFN-γ was secreted (Figure 1b, d).

**FIGURE 1:**
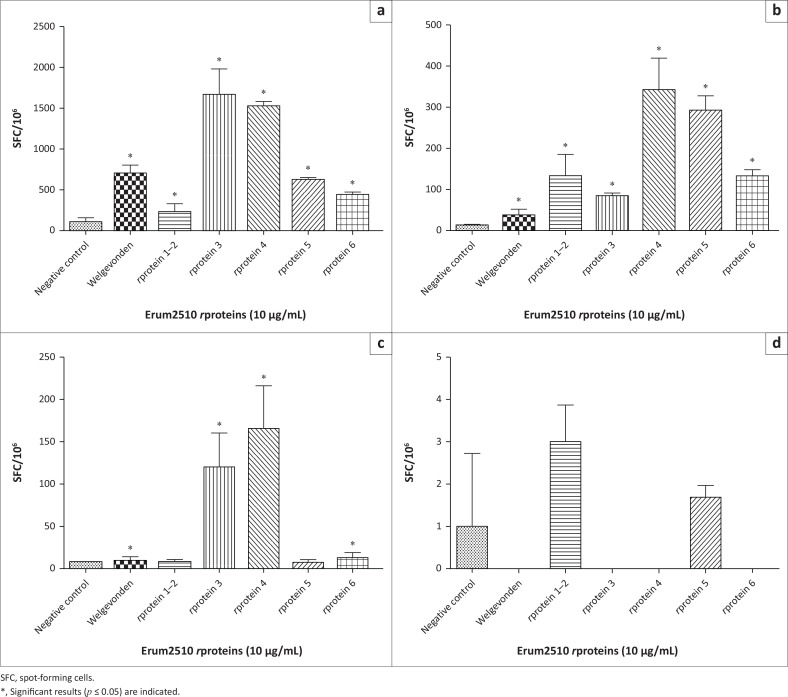
Secretion of interferon gamma (IFN-γ) (a, b) and interleukin 4 (IL-4) (c, d) by immune peripheral blood mononuclear cells (PBMC) from s6823 (a, c) and sM2 (b, d) stimulated with Erum2510 *r*proteins and crude Welgevonden-strain antigen (positive control) and unstimulated PBMC (negative control). Data are presented as the average number of spot-forming cells per million.

### Cytokine profiling with recombinant proteins

Erum2510 *r*proteins were screened for their ability to induce the expression of IL-1, IL-2, IL-4, IL-10, IL-12, IL-18, iNOS, TNF, TGF and GM-CSF (Table 1). The cytokine profile induced by the *r*Proteins in PBMC from both sheep was characterised by: *r*Protein 3 (IL‑1, IL-2, IL-10 and TNF), *r*Protein 4 (IL-1, IL-4, IL-10, IL-12, IL-18, TNF, TGF and GM-CSF), *r*Protein 5 (IL-1, IL-18 and TNF) and *r*Protein 6 (iNOS).

**TABLE 1:** Relative fold change in cytokine mRNA isolated from sM2 and s6823, stimulated with Erum2510 *r*proteins and normalised to endogenous reference genes glyceraldehyde 3-phosphate dehydrogenase (GAPDH) and β-actin, relative to an untreated negative control (unstimulated peripheral blood mononuclear cell).

Sheep ID	*r*Erum2510 ID	Cytokine mRNA fold change (GAPDH; β-actin)									
		IL‑1	IL‑2	IL‑4	IL‑10	IL‑12	IL‑18	iNOS	TNF	TGF	GM-CSF
s6823	1 & 2	0; 0	0; 1	0; 0	0; 0	0; 1	0; 0	1; 3	1; 0	0; 0	0; 0
	3	25; 67	3; 13	1; 5	7; 23	3; 0	1; 0	19; 22	19; 36	1; 5	1; 5
	4	367; 276	664; 128	389; 176	120; 232	611; 119	346; 98	1144; 678	1144; 225	24; 51	175; 57
	5	6; 9	1; 4	1; 0	2; 4	1; 11	2; 5	10; 3	10; 14	1; 10	1; 0
	6	1; 1	7; 0	1; 0	19; 1	7; 1	5; 0	81; 185	81; 0	2; 0	3; 0
	Negative control	1; 1	1; 1	1; 1	1; 1	1; 1	1; 1	1; 1	1; 1	1; 1	1; 1
sM2	1 & 2	11; 2	0; 0	1; 0	18; 4	2; 0	17; 4	1; 0	3; 1	38; 18	2; 7
	3	21; 22	2; 9	6; 10	10; 3	2; 0	31; 10	5; 1	10; 13	0; 1	10; 0
	4	10; 6	1; 1	2328; 1399	3; 2	13; 13	22; 13	4; 3	32; 67	4; 19	34; 11
	5	16; 5	4; 0	5; 1	7; 1	6; 2	18; 6	4; 0	8; 2	5; 1	11; 10
	6	14; 5	0; 0	3; 1	5; 0	2; 1	50; 22	4; 2	27; 8	11; 2	12; 1
	Negative control	1; 1	1; 1	1; 1	1; 1	1; 1	1; 1	1; 1	1; 1	1; 1	1; 1

mRNA, messenger ribonucleic acid; GAPDH, glyceraldehyde 3-phosphate dehydrogenase; IL, interleukin; iNOS, inducible nitric oxide synthase; TNF, tumour necrosis factor; TGF, transforming growth factor; GM-CSF, granulocyte-macrophage colony-stimulating factor.

### Phenotypic analysis with recombinant proteins

A statistically significant increase of the CD4+IFN-γ+ population in immune PBMC from s6823 was observed after incubation with Erum2510 *r*protein 4 (4.29%) and 6 (2.8%) (Figure 2a). Similar results were also observed in immune PBMC from sM2, *r*protein 4 (2.3%) and *r*protein 6 (1.5%) (Figure 2b). In contrast, none of the Erum2510 *r*proteins induced significant activation of CD8+IFN-γ+ population in immune PBMC from both sheep (Figure 2a, b). *r*Proteins 4 and 6 were also shown to induce statistically significant activation of memory CD4+ T lymphocytes in immune PBMC from s6823 (38.6%; 29.4%, respectively) while only *r*protein 6 induced a similar response in sM2 (24.9%) (Figure 4-A1). However, none of the Erum2510 *r*proteins induced statistically significant activation of memory CD8+ T lymphocytes (Figure 4-A1). Only *r*Protein 4 induced a statistically significant increase of the B+ population in immune PBMC from s6823 (Figure 3a).

**FIGURE 2:**
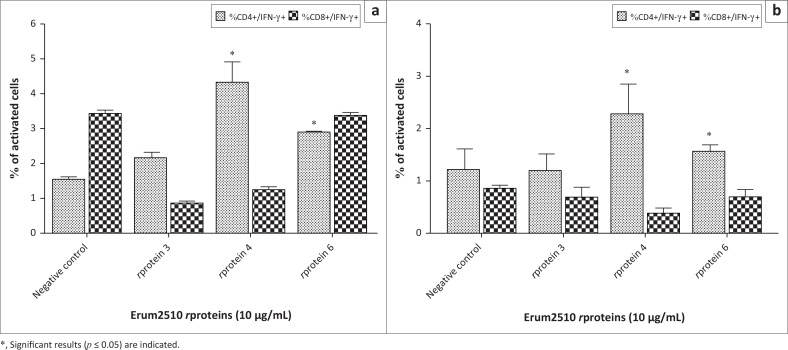
Average percentage of peripheral blood mononuclear cells (PBMC) from s6823 (a) and sM2 (b) that stained positive for CD4 and interferon gamma (IFN-*γ*) and CD8 and IFN-γ expression markers after stimulation with Erum2510 *r*proteins 3, 4 and 6. Unstimulated PBMC was included as negative control. Peripheral blood mononuclear cells were stimulated with 10 μg/mL *r*protein and incubated for 72 h, after which the percentage of cells was determined by flow cytometry.

**FIGURE 3:**
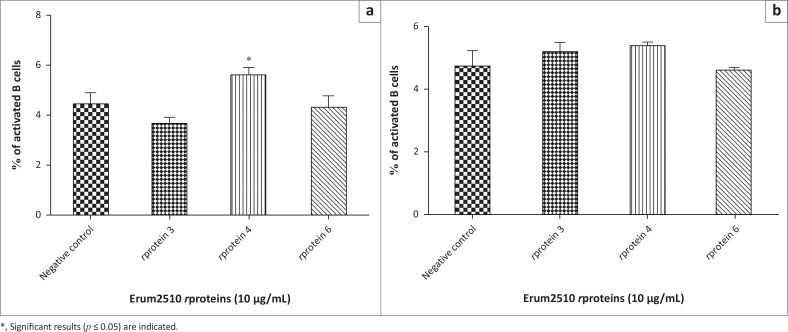
Average percentage of immune peripheral blood mononuclear cells (PBMC) from s6823 (a) and sM2 (b) that stained positive for B cell expression markers after stimulation with Erum2510 *r*proteins 3, 4 and 6. Unstimulated PBMC was included as negative control. Peripheral blood mononuclear cells were stimulated with 10 μg/mL protein and incubated for 72 h, after which the percentage of cells was determined by flow cytometry.

### Peptide synthesis

Data obtained from immunological assays carried out with five Erum2510 *r*proteins led to the conclusion that *r*proteins 3 and 4 were the most immunogenic. *r*Protein 4 was shown to be immunodominant, with an ability to induce cytokines associated with both Th1 and Th2 responses. Based on this, overlapping synthetic peptides were designed spanning the length of Erum2510 *r*proteins 3 and 4 and evaluated for their ability to induce recall immune responses.

### Lymphocyte proliferation assay with peptides

To identify immunogenic peptides of *r*proteins 3 and 4 that are recognised by immune PBMC from s6823 and sM2, all 37 peptides synthesised were individually screened in a LPA assay. Only four peptides from *r*protein 3 (p9, p10, p17 and p18) and four from *r*protein 4 (p24, p25, p28 and p29) induced statistically significant proliferation above the cut-off point in PBMC isolated from both sheep (Figure 5-A1). The proliferative response induced in sM2 PBMC by peptides was similar to those obtained with the *r*proteins 3 and 4. However, the proliferative responses induced in s6823 by peptides was shown to be slightly lower than those obtained by *r*proteins 3 and 4. These eight peptides were selected for further immune analysis.

### Enzyme-linked immunospot assay with peptides

Eight of the selected peptides from the LPA assay (10 μg/mL) were incubated in triplicate with immune PBMC isolated from sM2 and s6823 in an ELISpot assay. All eight peptides induced significant expression of IFN-γ in PBMC from both sheep, with the exception of peptide 29, which did not induce significant responses in s6823 (Figure 4). Only peptide 28 was shown to induce the expression of both IL-4 and IFN-γ from sM2 (Figure 4); however, no significant expression of IL-4 was observed for s6823. For further analysis, the eight peptides were pooled into four groups: pool A, *r*protein 3, p9 and p10; pool B, *r*protein 3, p17 and p18; pool C, *r*protein 4, p24 and p25; pool D, *r*protein 4, p28 and p29.

**FIGURE 4:**
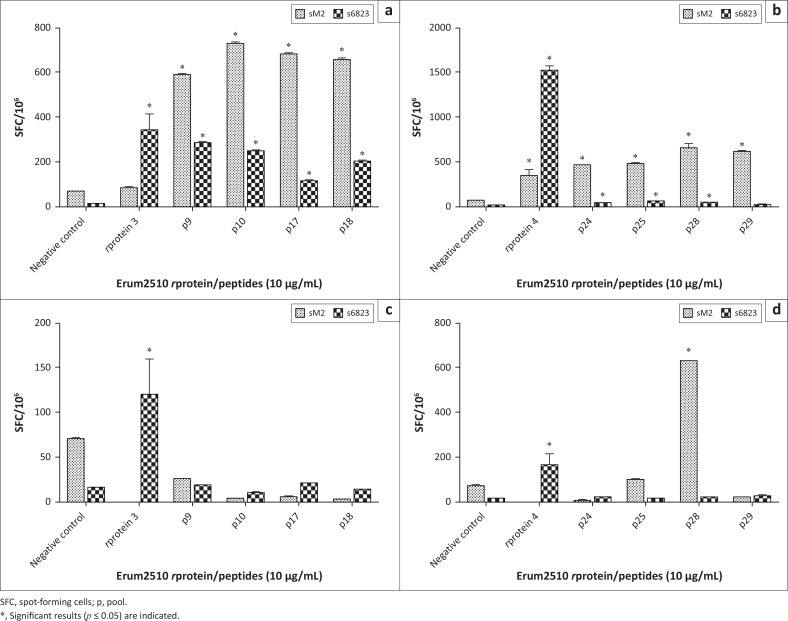
Secretion of interferon gamma (IFN-γ) (a, b) and interleukin 4 (IL-4) (c, d) by immune peripheral blood mononuclear cells (PBMC) from sM2 and s6823 stimulated with Erum2510 *r*proteins and peptides at a concentration of 10 μg/mL. Only those peptides with positive results are shown with (a, c) *r*protein 3 and corresponding peptides; (b, d) *r*protein 4 and corresponding peptides. Unstimulated PBMCs were included as negative control. Data are presented as the average number of spot-forming cells per million.

### Cytokine profiling with peptides

The mRNA cytokine fold expression profiles induced by the peptides were highly variable between sheep and are listed in Table 2. Although the values were generally higher when GAPDH was used as housekeeping gene, they did correlate with β-actin housekeeping gene results. In s6823 but not sM2, IL-4 and iNOS was induced when PBMC were stimulated with pools B, C and D. In sheep sM2 but not s6823, IL-1, TNF and TGFB were produced by all pools tested.

**TABLE 2:** Relative fold change in cytokine mRNA in peripheral blood mononuclear cells from sM2 and s6823 stimulated with rErum2510 synthetic peptides and normalised to endogenous reference genes glyceraldehyde 3-phosphate dehydrogenase (GAPDH) and β-actin, relative to an untreated negative control (unstimulated peripheral blood mononuclear cell).

Sheep ID	Erum2510 peptide pool ID	Cytokine mRNA fold increase (GAPDH; β-actin)									
		IL‑1	IL‑2	IL‑4	IL‑10	IL‑12	IL‑18	iNOS	TNF	TGF	GM-CSF
s6823	A	4; 2	0; 0	1; 4	0; 0	0; 0	1; 0	4; 1	0; 0	4; 1	0; 0
	B	0; 0	6; 1	2; 10	0; 0	0; 0	0; 0	21; 6	0; 0	0; 0	0; 0
	C	0; 0	13; 3	4; 6	0; 0	0; 0	0; 0	9; 2	0; 1	1; 0	0; 0
	D	1; 2	12; 2	3; 4	1; 0	0; 0	0; 0	11; 5	0; 1	0; 0	0; 0
	**Negative control**	1; 1	1; 1	1; 1	1; 1	1; 1	1; 1	1; 1	1; 1	1; 1	1; 1
sM2	A	14; 10	2; 1	0; 0	8; 2	5; 2	1; 0	1; 0	45; 12	135; 34	6; 2
	B	24; 8	3; 0	0; 0	10; 3	4; 1	3; 1	1; 0	49; 8	103; 65	2; 1
	C	18; 4	4; 1	0; 0	1; 0	7; 2	6; 2	2; 1	82; 32	102; 77	3; 0
	D	99; 24	3; 1	1, 0	6; 2	25; 5	6; 0	1; 0	42; 44	170; 45	6; 3
	**Negative control**	1; 1	1; 1	1; 1	1; 1	1; 1	1; 1	1; 1	1; 1	1; 1	1; 1

mRNA, messenger ribonucleic acid; GAPDH, glyceraldehyde 3-phosphate dehydrogenase; IL, interleukin; iNOS, inducible nitric oxide synthase; TNF, tumour necrosis factor; TGF, transformation growth factor; GM-CSF, granulocyte-macrophage colony-stimulating factor.

### Phenotypic analysis with peptides

To determine the population of lymphocytes responding to peptides, flow cytometry was carried out. Peptide pools A and C induced a significant increase of CD4+IFN-γ+ cells by PBMC from s6823 (Figure 5a). Peptide pools A, B and D induced a significant increase of CD4+IFN‑γ+ in sM2 (Figure 5b). In contrast to the *r*protein result, peptide pool D induced a significant increase of CD8+IFN-γ+ cells by PBMC from both sheep (Figure 5). Peptide pools A, B, C and D induced a significant activation of memory CD4+ T lymphocytes (Figure 6-A1). In contrast to results obtained from Erum2510 *r*proteins, all peptide pools (A, B, C, D) induced a significant increase of memory CD8+ T cells by immune PBMC from both sheep. Peptide pool D induced a statistically significant increase of B cells in both sheep (Figure 6).

**FIGURE 5:**
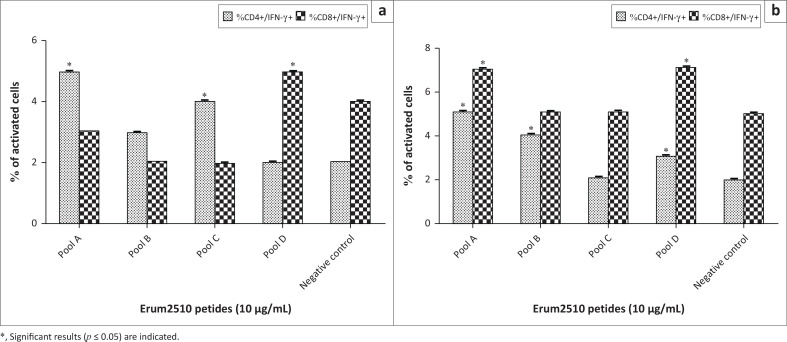
Average percentage of peripheral blood mononuclear cells (PBMC) from s6823 (a) and sM2 (b) that stained positive for CD4 and interferon gamma (IFN-*γ*), and CD8 and IFN-*γ* expression markers after stimulation with Erum2510 peptide pools. Peripheral blood mononuclear cells were stimulated with 10 μg/mL protein and incubated for 72 h, after which the percentage of cells was determined by flow cytometry. Unstimulated PBMCs were included as negative control.

**FIGURE 6:**
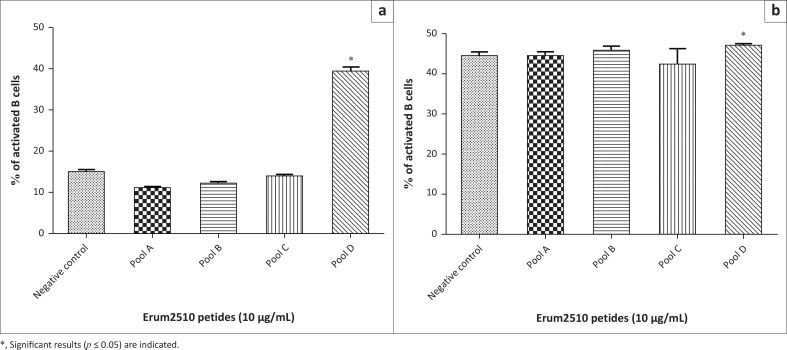
Average percentage of peripheral blood mononuclear cells (PBMCs) from s6823 (a) and sM2 (b) that stained positive for B cell expression markers after stimulation with Erum2510 peptide pools. Peripheral blood mononuclear cells were stimulated with 10 μg/mL protein and incubated for 72 h, after which the percentage of cells was determined by flow cytometry. Unstimulated PBMCs were included as negative control.

## Discussion

*Cowdria* polymorphic gene 1 (*cpg1*, Erum2510) was previously shown to induce lymphocyte proliferation, antibodies and protection against heartwater in sheep using a DNA prime and *r*protein boost immunisation strategy (Pretorius et al. 2010). This implicates Erum2510 as a potential vaccine candidate for inclusion in an epitope-based vaccine against heartwater. To identify its antigenic epitopes, Erum2510 was divided into six overlapping subfragments. Subfragments 1 and 2 were cloned as a single fragment and subfragments 3–6 were cloned individually to produce five *r*proteins. Erum2510 *r*protein 3 and 4 induced proliferation of PBMC obtained from both sM2 and s6823, with the strongest response observed in s6823. In contrast, all the Erum2510 *r*protein subfragments induced significant expression of IFN-γ in PBMC from both sheep quantified in the ELISpot assay. However, significant expression of IL-4 was induced by *r*protein 3 and 4, only in s6823 PBMC.

The expression of additional cytokines associated with protection by immune PBMC in response to Erum2510 *r*proteins was investigated by qRT-PCR. Erum2510 *r*protein 4 induced strong expression of IL-2 and TNF-α (Th1) mRNA, accompanied by strong expression of IL-10 and IL-4 (Th2) mRNA. These results thus correlate with the ELISpot assay for sheep s6823. However, there were differences observed in this study between gene expression and protein levels detected in PBMC from sM2 in response to *r*protein 4. The overproduction of IL-4 mRNA induced by this *r*protein may have activated mRNA post-transcriptional expression regulation, which includes mRNA degradation mechanisms in order to avoid the detrimental effects caused by the overproduction of IL-4 protein (Junttila 2018; Wierenga & Messer 2000). Although the mechanism of the observed regulation is not known, RNA decay can be mediated by RNA decay–promoting proteins, namely BRF1, KSRP and AUF1, which was not tested for in the current study (Khabar 2007). This highlights the importance of comparison between mRNA expression levels and protein cytokine secretion levels, as the detection of mRNA does not necessarily mean that the protein is produced (Shebi et al. 2010). Thus, conclusions derived from cytokine mRNA levels alone should be made with caution unless protein expression can be verified.

Erum2510 *r*protein 4 induced significant proliferation of B+ lymphocytes, CD4+CD45R0+ T cells and CD4+IFN-γ+ T cells. None of the Erum2510 *r*proteins assayed were shown to induce significant activation of CD8+ T cells in both sheep. Results therefore indicate that in both sheep, the cytokine profile induced by Erum2510 *r*protein 3 and 4 is characteristic of a Th1 and Th2 mixed immune response. The expression of IL-4 and IFN-γ mRNA strongly suggest that *r*proteins 3 and 4 contain Th1 and Th2 (B cell) epitopes that could induce a cellular and humoral response against *E. ruminantium*. Combined Th1 and Th2 responses induced by a *r*protein have been described (Daifalla, Bayih & Gedamu 2015; Stachyra et al. 2019). For example, mice vaccinated with the recombinant saposin-like protein FhSAP-2, from *Fasciola hepatica*, induced high detectable levels of IgG1, IgG2 and IgE, as well as high levels of IL-10 and IFN-γ indicative of a mixed Th1 and Th2 response (Espino et al. 2005). Thus, the induction of Th1 and Th2 mixed responses to pathogen antigens is not uncommon and is beneficial to the immune response. The similar strong co-expression of Th1 and Th2 cytokine mRNA observed suggests that Erum2510 *r*proteins 3 and 4 contain both Th1 and Th2 epitopes, possibly working in concert to eliminate infectious agents and ensure control of the immune response.

Because Erum2510 *r*protein 3 and 4 were shown to be the most immunogenic, they were divided into 37 overlapping synthetic peptides to identify specific epitopes. The immune response induced by the synthetic peptides was firstly characterised by LPA assay. Eight out of 37 peptides induced PBMC from both animals to proliferate. These eight peptides (*r*protein 3: p9, p10, p17, p18; *r*protein 4: p24, p25, p28, p29) were then screened with ELISpot, and they all induced statistically significant expression of IFN-γ in PBMC isolated from both sheep, as did their respective *r*proteins. Only peptide 28, derived from *r*protein 4, induced statistically significant expression of IL-4 in sM2, whereas the *r*protein did not. This may indicate that there are epitopes in the *r*protein that suppress the immune response induced by the peptide, thus emphasising the importance of epitope mapping. Furthermore, no peptide induced expression of IL-4 in PBMC from s6823, while both *r*protein 3 and 4 did. The ability of peptide 28 to induce expression of IFN-γ and IL-4 suggests that it is a Th1 and Th2 epitope. Previous studies have similarly reported on the ability of a peptide to induce Th1 and Th2 responses (Chaturvedi et al. 1996; Wong et al. 2004).

Selected peptides were then pooled for qRT-PCR and flow cytometry assays. Peptide pool A (*r*protein 3: p9, p10) induced a Th1-biased response characterised by the expression of IL-12, TNF and GM-CSF. Peptide pool D (*r*protein 4: p28, p29) induced a Th1 and Th2 response characterised by the expression of IFN-γ, IL-2, TGF and IL-4 (p28). Phenotypic analysis showed that peptide pool A (rprotein 3: p9, p10) and peptide pool C (*r*protein 4: p24, p25) activated a significant percentage of CD4+ T cells expressing IFN-γ in s6823 PBMC. Interestingly, all peptide pools were able to induce significant activation of CD8+CD45R0+ T cells and CD4+CD45R0+ T cells. A higher percentage of CD8+CD45R0+ T cells than CD4+CD45R0+ T cells was noted for peptide pool A (*r*protein3: p9, p10) and peptide pool D (*r*protein 4: p28, 29) in both sheep. This contrasts with the data obtained from Erum2510 rproteins, wherein none of the *r*proteins were shown to activate CD8+CD45R0+ T cells. This may be because the rproteins were not processed correctly or that they contained other epitopes that interfered with the CD8+ T cell response (Wang et al. 2005).

The variability in response of the two sheep to the same antigen observed in the study can be attributed to the usage of outbred animals (genetic variability), which has been reported by other groups (Esteves et al. 2004; Thema et al. 2019a, 2019b). Major histocompatibility complex (MHC) typing of sM2 (results not shown) and s6823 (Thema et al. 2016) showed different ovar-DRB1 alleles at exon 20, s6823: *0201, 0801 and sM2: *0323, 0333. Although these preliminary findings indicated that the peptides produced a ‘weaker’ response in s6823 compared with sM2, they have the potential to induce an immune response associated with protection. Therefore, they should be further investigated as vaccine candidates, using larger statistically significant groups of sheep, goats and cattle.

In conclusion, multi-epitope vaccines containing both cellular and humoral epitopes induce strong and often enhanced immune responses, as shown in studies on *Plasmodium falciparum*, malaria (Shi et al. 2000), human immunodeficiency virus (HIV) (Karpenko et al. 2007) and herpes simplex virus (Wang et al. 2011), to name a few. The ability of Erum2510 *r*protein 4 and synthetic peptides (p28, p29) to induce statistically significant Th1, Th2 and B cell responses in this study, as well as antibodies directed to *r*Erum2510 and protection of sheep in a previous study (Pretorius et al. 2010), merits investigating them as vaccine candidates in a multi-epitope vaccine against heartwater.

## Figures and Tables

**FIGURE 1-A1:**
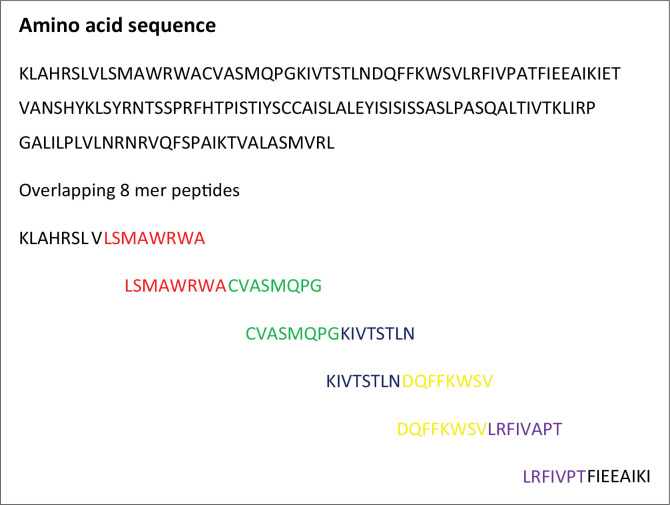
Schematic representation of overlapping peptides spanning the length of Erum2510 Fragment 4 used for epitope mapping. Overlapping peptides used in this study were designed in the same format.

**FIGURE 2-A1:**
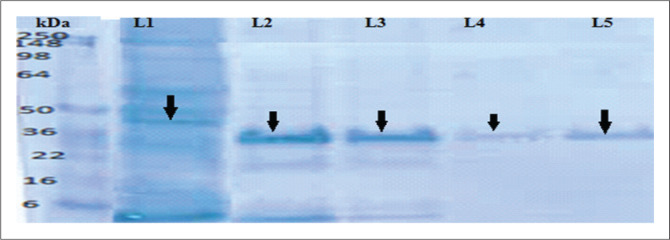
Anti-His_6_ Western blot analysis illustrating purified recombinant proteins (indicated with arrows). (kDa: Molecular weight marker with size indicated in kDa; L1: sub-fragment 1-2, L2: sub-fragment 3, L3: sub-fragment 4, L4: sub-fragment 5, L5: sub-fragment 6).

**FIGURE 3-A1:**
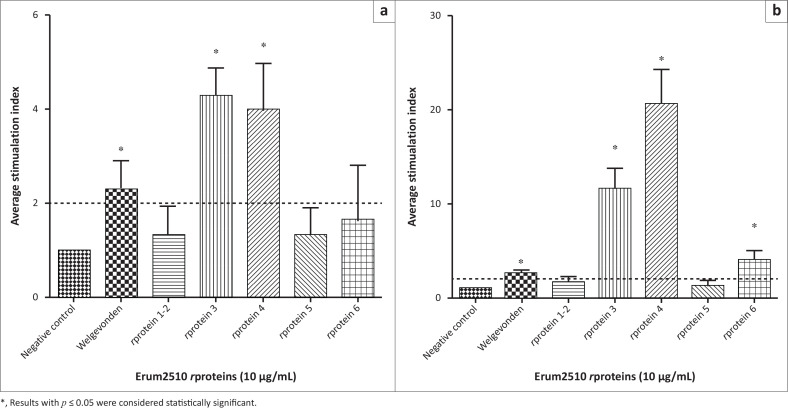
Average stimulation index (SI) indicating proliferative response of immune peripheral blood mononuclear cells isolated from sM2 (a) and s6823 (b) to five Erum2510 sub-rproteins. Crude Welgevonden strain antigen was included as a positive control and unstimulated peripheral blood mononuclear cells as negative control. Horizontal dashed line represents a stimulation index of 2, which is the cut-off point for a positive response.

**FIGURE 4-A1:**
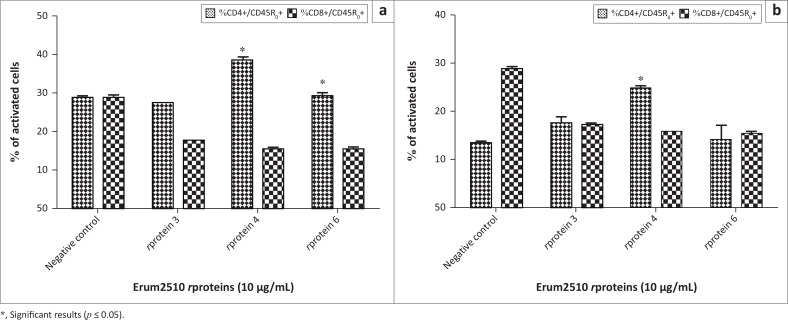
Average percentage of peripheral blood mononuclear cells from s6823 (a) and sM2 (b) that stained positive for CD4 and CD45RO; and CD8 and CD45RO expression markers after stimulation with Erum2510 rproteins 3, 4 and 6. Unstimulated peripheral blood mononuclear cells was included as negative control. peripheral blood mononuclear cells were stimulated with 10 μg per ml protein and incubated for 72 h after which the percentage of cells was determined by flow cytometry.

**FIGURE 5-A1:**
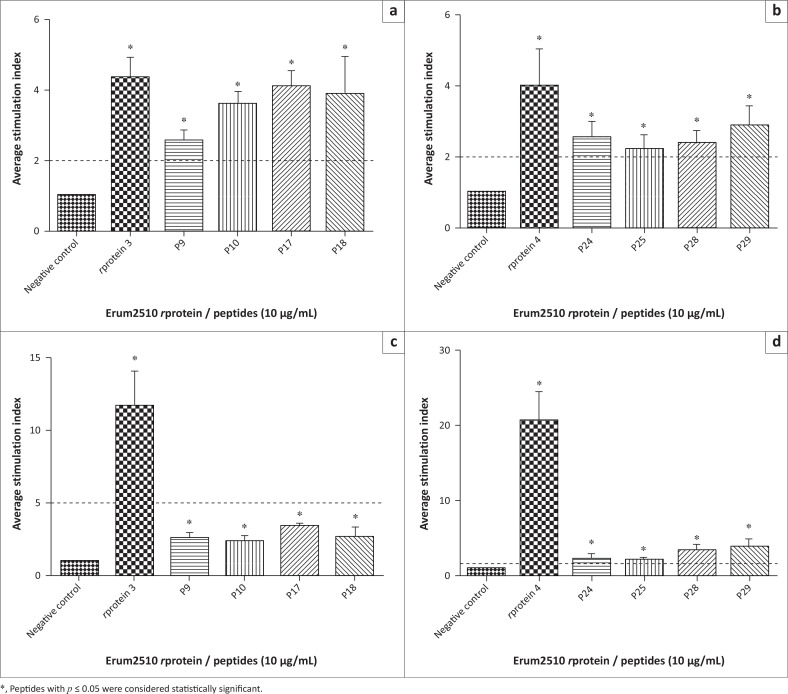
Average stimulation index of immune peripheral blood mononuclear cells isolated from sM2 (a, b) and s6823 (c, d) after incubation with Erum2510 rproteins and peptides. (a, c) rprotein 3 and corresponding peptides; (b, d) rprotein 4 and corresponding peptides. Unstimulated peripheral blood mononuclear cells were included as negative control. The horizontal dashed line represents a stimulation index of 2, which is the cut-off point for a positive response.

**FIGURE 6-A1:**
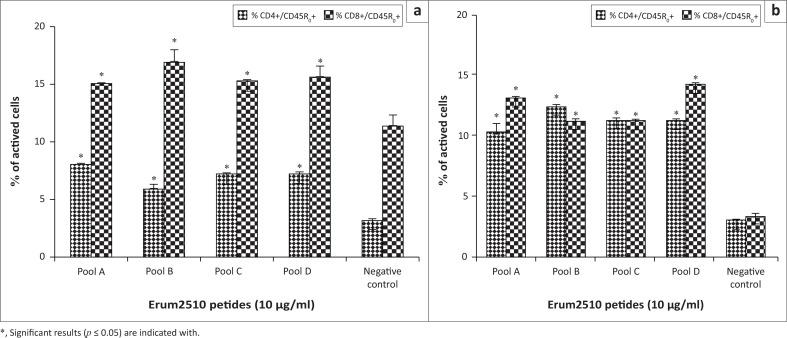
Average percentage of peripheral blood mononuclear cells from s6823 (a) and sM2 (b) that stained positive for CD4 and CD45R0, and CD8 and CD45R0 expression markers after stimulation with Erum2510 peptide pools. peripheral blood mononuclear cells were stimulated with 10 μg/mL protein and incubated for 72 h after which the percentage of cells was determined by flow cytometry. Unstimulated peripheral blood mononuclear cells were included as negative control.

## Appendix 1

**TABLE 1-A1:** Rotor-gene Q ovine quantitative real time polymerase primers used to quantify cytokine mRNA targets in quantitative real time polymerase chain reaction assay.

Target	Forward primer 5ʹ-3ʹ	Reverse primer 5ʹ-3ʹ
**IL-1α**	TTG GTG CAC ATG GCA AGT G	GCA CAG TCA AGG CTA TTT TTC C
**IL-4**	TGA CAG GAA TCT CAG CAG	TTC TCC CTC ATA ATA GTC TTT AGC
**IL-10**	TGG AGC AGG TGA AGA GAG TCT	AAA CTC ACT CAT GGC TTT GTA GAC A
**IL-12**	ATC GTG GCC ATA TGG GAA CTG	CTC CAG GAG CAT TAG GAT ACC AATC
**IL-18**	CCT GTC TTT GAG GAT ATG CCT GAT T	GGT TAC AGC CAG ACC TCT AGT GA
**TNFα**	CCC AGG GCT CCA GAA GTTG	GCA ACC AGG AGG AAG GAG AA
**TGFβ**	GAA CTG CTG TGT TCG TCAG C	GGT TGT GCT GGT TGT ACA GG
**GAPDH**	TCA CTG CCA CCC AGA AGA	CTC AGG GAT GAC CTT GC
**B-actin**	GCT CTT CCA GCC GTC CTT	TGA AGG TGG TCT CGT GAA TGC
**IL-2**	TCT ACA TGC CCA AGG TTA ACG	CTA CAG CCT TTA CTG TCG C
**INOS**	ATC TGC AGA CAC GTG CGT TA	GTT CCA GAC CCG GAA GTC AT
**GM-CSF**	ATG GAT GAA ACA GTA GAA GTC G	CAG CAG TCA AAG GGA ATG AT
